# Study on the Relationship between Nurses’ Mentoring Relationship and Organizational Commitment

**DOI:** 10.3390/ijerph192013362

**Published:** 2022-10-16

**Authors:** Zhenxing Gong, Lyn M. Van Swol, Xiangge Wang

**Affiliations:** 1 School of Business, Liaocheng University, Liaocheng 252000, China; 2 Department of Communication Arts, University of Wisconsin—Madison, Madison, WI 53706, USA

**Keywords:** mentoring relationship, organizational commitment, protégé career optimism, protean career orientation

## Abstract

The mentoring relationship affects the growth and development of new employees. For nurses, the uncertainty of the influence of the mentoring relationship may be magnified by the unique nature of hospitals as public departments, however it is unclear whether and how nurses’ mentoring relationship influence the outcome. Protean career orientation defined as a tendency of individuals to achieve subjective career success through self-management of their career is crucial to the influence mechanism of the mentoring relationship. The aim of this study was to explore the path and boundary conditions of the influence of the nurses’ mentoring relationship on organizational commitment. As a cross-sectional sample, 371 nurses were investigated. The results showed that protégé career optimism plays an intermediary role in the influence of the mentoring relationship on organizational commitment, and protean career orientation plays a moderating role in the influence of the mentoring relationship on career optimism. The mentor relationship between mentors and protégés facilitates protégés’ career optimism, enhancing the protégés’ organizational commitment, especially for protégés with low protean career orientation. These findings contribute to the improving nurses’ organizational commitment through mentoring relationship. Hospitals should provide space for nurses to exert their abilities, enhance opportunities to improve their team cooperation ability, clearly define the scope of nurses’ work and rights, and give nurses the right to make decisions.

## 1. Introduction

The turnover intention of nurses has an impact on patients’ health and the stable development of the hospital during delivery. The turnover intention of new nurses is usually higher than that of older nurses [1]. Studies show that the turnover rate of nurses in the first year of internship is between 35% and 60% [2]. Because of the high turnover rate of new nurses, hospitals need to take certain management measures to prevent and solve the problem of brain drain. Organizational commitment is an individual’s recognition and trust of the goals and values of the organization to which he or she belongs and the positive emotional experience brought about by it. New nurses lack clinical nursing experience, have limited nursing skills, and must take care of patients. Job stress leads to turnover intention. However, research shows that improving organizational commitment can reduce the turnover intention of new nurses [3]. Therefore, to reduce nurses’ turnover intention, hospital administrators urgently need to improve the organizational commitment of new nurses.

Mentoring relationship is defined as the close interaction between older and more experienced people and younger and less experienced people [4]. The mentorship has been used to help new employees enter and adapt to the workplace. Senior nurses help new nurses become familiar with their work by imparting nursing-related policies and procedures, help new nurses improve their skills by working together, and provide social and emotional support and role models in the course of work, which are the key components of the induction process [5]. A good mentoring relationship is conducive to the professional and personal growth of protégés, such as helping protégés to be promoted, providing effective strategies to reach their goals, and providing opportunities for protégés to show their abilities. Regardless of psychological or spiritual support, protégés can obtain help from their mentors. The research shows that compared with their peers who do not get a mentor, protégés who have a mentor’s guidance gain more organizational and professional benefits. According to social exchange theory, protégés should repay the guidance of the organization and the mentor (for example, by increasing commitment).

However, regarding the outcomes of the mentoring relationship, there are some knowledge gaps we need to explore. First, most of the research focuses on the research of objective outcomes, such as salary or promotion [6], rather than more intrinsic problems, such as organizational commitment. Second, most studies focus on the positive role of the mentoring relationship in private companies [7]. In recent years, some studies have explored the negative effects of the mentoring relationship, such as role confusion, interpersonal conflict, and lack of individual rights and dilution of organizational culture [8]. Due to the low flexibility of incentive policies and budget constraints, the implementation of a public mentorship will have a negative impact on the mentoring relationship due to mere formality or lack of responsibility for behaviors outside the role [9]. The uncertainty of the result of the mentoring relationship may be amplified by the unique nature of the hospital in the public sector. Third, it is unclear under what conditions mentoring is more likely to promote commitment. The aim of this study was to explore the path and boundary conditions of the influence of the nurses’ mentoring relationship on organizational commitment.

Although the theory of mentoring and professional cognition recognizes the importance of individual cognition, researchers also call for a systematic investigation of the role of cognitive factors [10], and there are still few empirical studies. However, the empirical research on the management of the mentoring relationship of new nurses is more important because the new nurses’ predictability of career development is reduced due to their heavy responsibilities, heavy tasks and little experience. Individuals are to become more flexible to adapt to career development and job changes, and the benefits of the mentoring relationship are limited if they are separated from their concern for future career development and self-management.

Cognitive career theory emphasizes the influence of personal cognition, background and learning on career choice behavior. Protean career orientation is a tendency for individuals to achieve subjective career success by independently managing their careers, which has the dual attributes of being self-oriented and values-driven [11]. Career optimism is an individual’s expectation and a positive attitude toward their best future career development [12]. Career obstacles are only temporary for individuals who are optimistic about their career, who tends to prepare for the imaginary future and feel that they are on the way to career success.

In view of the environmental differences between the public sector and private enterprises, the present study contributes to the literature in several ways. First, we fill an existing research gap by explaining the relationship between mentoring relationships with organizational commitment for nurses. Second, we investigate the mediation role of protégé’s career optimism, which provides a promising opportunity to discover the mechanism of the mentoring relationship influence on organizational commitment. Third, a primary aim of the present study, accordingly, was to investigate under what condition is the mentoring relationship more likely to promote organizational commitment. To do so, we explore the moderating role of nurses’ protean career orientation.

## 2. Theory and Hypotheses

### 2.1. Mentoring Relationship and Organizational Commitment

To provide protégé career development and psychological support, role models promote the exchange of knowledge and experience between the senior mentor and junior protégé, improve the protégé’s ability, bring growth and profit opportunities to the organization, and promote its continuous growth [13]. Career guidance focuses on preparing for the protected person’s career development, role shaping focuses on conveying appropriate attitudes, values and behaviors, and social support focuses on providing positive attention and practical help. Employees are expected to repay the interests of the organization related to supervision and guidance. Organizational commitment refers to an individual’s recognition and participation in a specific organization [6]. The antecedents of organizational commitment include positive relationships with colleagues and supervisors, opportunities for feedback, and interactions with effective social agents [14]. The mentor often provides feedback as the protégé’s colleague or supervisor and helps the protected person to adapt to the norms of the organization [4]. The research shows that the mentor’s guidance can help the protégé establish a sense of belonging to the organization and a sense of community and feel that he or she is accepted by the organization. The mentor is the intermediary between the organization and the protégé, which can increase the sense of commitment of both mentors and protégés to the organization [8]. The mentoring relationship itself is a kind of communication relationship that can make the news between the two departments represented by the mentor and protégé circulation, and the mentor and protégé can have a close emotional connection with the organization [4]. Passing on the value, purpose and tradition of organizational culture through the guidance of the mentor can increase the protégé’s sense of identity with the organization.

**Hypothesis** **1.** *The mentoring relationship positively influences the organizational commitment of the protégé* **.**

### 2.2. The Mediation Role of Career Optimism 

The three functions of the mentoring relationship can improve protégés’ career optimism. Specifically, the function of vocational support emphasizes individualized consideration and attention to the unique needs of each person. The mentor can help the protégé acquire more useful skills and knowledge, and the individualized care given enables the protégé to adapt to the working environment as soon as possible [15]. Social psychological support enables protégés to be recognized by their mentors. By clarifying a convincing vision, the mentor conveys the expectation and confidence of the protégé for high performance [16]. The role model function allows the mentor to serve as an example for the protégé and promotes their development by conveying their confidence [15]. The role model can show the charm and idealized influence of the mentor, and the obvious goal is passed on to the protégé through the values, behaviors and achievements of the role model [16].

The mentoring relationship emphasizes the dynamic process of the mentor and protégé learning together. Career optimism may include the expectation of favorable results, even though unfavorable consequences may occur [12]. Career optimism people can deal with stress effectively [17]. When a career-optimism individual adapts to an uncertain environment, he thinks that although his career choice is negatively affected, his achievements in the organization and the acceptance of the members of the organization make him think it is worth staying in the organization [12]. Career optimism individuals spend more energy on achieving their career goals, so they maintain this effort with a greater sense of career optimism, which may lead to greater organizational commitment [17].

The mentoring relationship can increase the stickiness of the mentor and protégé so that protégés can calmly look at their own success and increase their organizational commitment [4]. When providing guidance and consultation and asking the protégé to complete the main tasks on time, the mentor creates a vision of success and inspires the protégé to acquire knowledge and skills [18]. When the mentor provides challenging tasks and advice to the protégé, the tutor will stimulate the protégé’s curiosity and increase the chances of retaining him or her as an employee [19]. Mentors provide psychological and social support and guidance for protégés so that protégés can quickly integrate into the organization, adapt to their jobs and roles, be full of hope for their future career development, and gain higher organizational commitment [18].

**Hypothesis** **2.** *Career optimism plays a mediation role in the influence of the mentoring relationship on organizational commitment* **.**

### 2.3. The Moderating Effect of Protean Career Orientation 

New employees want the organization to involve them in decision-making, provide challenging and meaningful jobs, support skills development, and provide career management assistance [20]. The popularity of the concept of protean career orientation further shows that employees usually regard organizational members as a tool through which they can achieve valuable personal goals. Protean career orientation is a relatively stable career preference. Protean career orientation attaches importance to self-orientation and defines career success according to personal values, i.e., it has the dual attributes of being self-oriented and values-driven [21]. Self-orientation emphasizes an individual’s independent behavior in the process of his or her career development, while values-driven orientation emphasizes the importance of individual intrinsic values in career choices [22].

Protean career orientation is an important factor that affects employees’ career development and work life. Individuals with higher career optimism are able to adapt to the changing professional environment [23]. Individuals with a highly protean career orientation tend to set clear career goals, make detailed career plans, and choose careers according to their own values. A career that is consistent with their values is more likely to make them feel successful. Individuals with a highly protean career orientation subjectively seek self-development. As a supplement to external resources, the mentoring relationship has little influence [21]. However, individuals with low protean career orientation find it difficult to face the changing economic environment because of their passive adaptation to the environment, and their career development path is unclear. The multiple functions of the mentoring relationship provide a better supplement for clarifying the career development path. Therefore, for individuals with low career orientation, the mentoring relationship can help protégés reduce frustration, improve their success rate and productivity, enhance their job satisfaction and overall life happiness, and increase their work engagement and loyalty. The mentor can help the protégé develop their professional knowledge, stimulate their potential interest in a certain subject, provide them with appropriate challenges, and guide them to develop their own interests and lifelong careers.

Protean career orientation is closely related to career planning and career goal development. Li and colleagues found that college students with high protean career orientation are more willing to obtain career-related information and resources [21]. People with a highly protean career orientation tend to define career success by their own standards, take actions to meet those standards and have higher career aspirations [23]. Therefore, they tend to take the initiative to find a more suitable working environment, which will weaken their positive role in organizational commitment.

**Hypothesis** **3.** *When protégé protean career orientation is low, the mentoring relationship has a great positive impact on protégé career optimism*.

**Figure 1:**
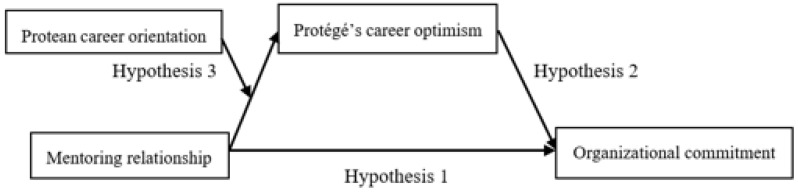
Test Model.

In sum, the goal of the present study was to understand how mentoring relationship impact on organizational commitment, test the mediation role of career optimism and the moderation role of protean career orientation. The moderated mediation model showed in Figure 1.

## 3. Materials and Methods

### 3.1. Study Design

The study included 371 first-line nurses in China as samples, using a cross-sectional design. According to the sampling method, which is convenient for sampling, the hospital that implements the mentoring relationship is taken as the place of data collection, and the cluster random sampling procedure is adopted to obtain representative samples.

### 3.2. Sample and Setting

The samples were taken from six regional general hospitals in China. We contacted participants through human resource managers of these hospitals. The study included regular nurses and interning nurses who had been instructed by their mentors for at least one month as protégé samples. These samples are guided by a mentor where they are working for at least 5–8 h every week. After explaining the aim, process and result of the study were not used for any purpose other than research, nurses were asked to participate in the study voluntarily and all surveys were processed anonymously together during working hours.

Finally, 371 valid questionnaires were obtained, which met the statistical efficacy standard (more than 136), and the effective rate was 82%. Among them, there were 43 males, accounting for 11.6%, and 328 females, accounting for 88.4%. The age of the sample is 23.78±2.53 years old, including 92 (24.8%) senior middle school students, 233 (62.8%) junior college students and 46 (12.4%) undergraduate students.

### 3.3. Measures

This study involves four variables, namely, mentoring relationship, career optimism, protean career orientation and organizational commitment. All four variables are measured by a 5-point Likert scale, with 1 representing total disagreement and 5 representing absolute agreement (see Appendix A). These questionnaires were shown in following order according to the order of influence mechanism.

#### 3.3.1. Mentoring Relationship

The mentoring relationship questionnaire revised by Hu et al. [24] includes three dimensions, career guidance, psychosocial support and role models, with nine items, such as “My mentor has devoted time and energy to my career”. Cronbach’s α of mentoring relationship is 0.96.

#### 3.3.2. Protean Career Orientation

The scale developed by Briscoe et al. [22] includes 14 items, of which 8 items measure self-directed career orientation and 6 items measure values-driven career orientation. Cronbach’s α of the overall scale is 0.97.

#### 3.3.3. Career Optimism

The 11-item career optimism scale compiled by Rottinghaus, Day and Borgen [25] was adopted. The sample titled “I am eager to achieve my career ideal” has a Cronbach’s α of 0.96.

#### 3.3.4. Organizational Commitment

A simplified version of the work commitment scale developed by Mowday, Porter and Steers [26], which consists of 9 items, was adopted. The Cronbach’s α of this scale is 0.98.

#### 3.3.5. Control Variables

In this study, demographic variables such as gender, age, length of service and educational level of employees are classified as control variables and controlled in the statistics because prior research has found demographic variables can influence the results of mentoring relationships [4].

### 3.4. Data Collection

Before the questionnaire survey, the consent of the hospital was achieved. Then, eligible samples were informed of the purpose and significance of this research and the principles of voluntariness and anonymity. The research results were kept confidential and used only for scientific research. The questionnaire was completed during working hours. Before the investigation, the researcher was approved by the university’s ethics committee.

### 3.5. Data Analysis

Mplus software was used to conduct confirmatory factor analysis of variables for testing construct validity, and SPSS 24.0 was used to conduct descriptive statistical analysis, correlation analysis, mediation analysis (Hypotheses 1 and 2) and moderation analysis (Hypothesis 3). *p* < 0.05 was used as the standard to evaluate the significance level.

## 4. Results

### 4.1. Validity Test of Variable Discrimination

To test the validity of discrimination between variables, this study uses Mplus software to conduct confirmatory factor analysis on the mentoring relationship, protean career orientation, career optimism and organizational commitment. Table 1 shows that the four-factor model has the best fitting validity (*x*^2^ = 37.112, *p* < 0.01, CFI = 0.996, TLI = 0.993, RMSEA = 0.035, SRMR = 0.013) compared with the single-factor, two-factor and three-factor models, which indicates that the four variables involved in the research model have good discrimination validity.

### 4.2. Descriptive Statistics and Correlation Analysis

The analysis results of the means, standard deviations and correlation coefficients of the related variables are shown in Table 2. According to the table, there is a significant positive correlation between mentoring relationship and career optimism (*r* = 0.64, *p* < 0.01), protean career orientation (*r* = 0.53, *p* < 0.01) and organizational commitment (*r* = 0.56, *p* < 0.01). There also are significant positive correlations between career optimism and organizational commitment (*r* = 0.49, *p* < 0.01) and between protean career orientation and career optimism (*r* = 0.66, *p* < 0.01).

### 4.3. Analysis of Direct, Mediation, Moderation and Conditional Process

In Table 3, mentoring relationship has a positive relationship with career optimism (*β* = 0.67, *p* < 0.01). In addition, there is a significant positive relationship between career optimism and organizational commitment (*β* = 0.25, *p* < 0.01). There is a significant positive relationship between the perceived mentoring relationship and the direct effect of organizational commitment (*β* = 0.49, *p* < 0.01). Hypothesis 1 is verified.

To test the mediation hypothesis, this research used a bootstrap mediation method with 5000 samples with replacement and percentile bootstrap confidence intervals. The mediating effect of the mentoring relationship on organizational commitment through career optimism is 0.17. The confidence interval of the bootstrap results is written BootLLCI (lower level for confidence interval) = 0.08 and BootULCI (upper level for confidence interval) = 0.28. The confidence interval does not contain zero, so the mediation effect is significant. Hypothesis 2 is verified.

In Table 4, When the protean career orientation is one standard deviation lower than the average, the indirect effect of mentoring relationship on organizational commitment through career optimism is 0.49 (confidence interval is [0.39, 0.59]). When the protean career orientation is one standard deviation higher than the average, the indirect effect of mentoring relationship on organizational commitment through career optimism is 0.34 (confidence interval is ([0.23, 0.44]). The interaction between mentoring relationship and protean career orientation has a significant effect on career optimism (*β* = −008, *p* < 0.01), which indicates that protean career orientation plays a moderating role between mentoring relationship and career optimism.

**Figure 2:**
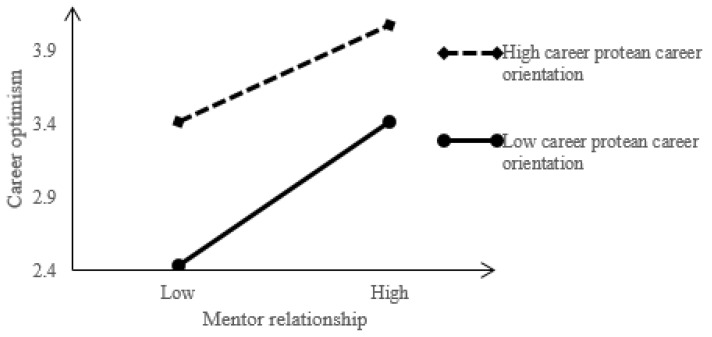
The moderating effect of protean career orientation on mentoring relationship-career optimism.

In addition to the analysis of the conditional indirect effects, protean career orientation has a moderating effect on the indirect relationship between mentoring relationship and organizational commitment, and the judgment index is −0.02 (confidence interval is [−0.04, −0.01]). As shown in Figure 2, for the group with low protean career orientation (1 standard deviation below the average), the positive relationship between mentoring relationship and career optimism is significant, while for the group with high protean career orientation (1 standard deviation above the average), this relationship is obviously weak. Therefore, compared with the employees with low protean career orientation, when the protean career orientation is high, the mentoring relationship has a great positive impact on career optimism. Hypothesis 3 is verified.

## 5. Discussion

The investigation of the mentoring relationship and organizational commitment in this study will not only help to clarify the influence path of the mentoring relationship on organizational commitment but also explain the reasons for the unstable positive effect of the mentoring relationship. The results show that the mentoring relationship influences organizational commitment through protégé career optimism, and the protean career orientation plays a moderating role in the mentoring relationship’s effect on career optimism.

All hypotheses in this study are supported. First, the mentoring relationship has a significant positive effect on the protégé’s organizational commitment. Consistent with previous studies [27], the research results support that the mentor, as an important source of knowledge transmission and continuity within the organization, can help with protégés’ career development and organizational goals and then improve the organizational commitment of protégés. A higher level of organizational commitment is inversely proportional to employee turnover, tardiness and absenteeism and directly proportional to work motivation and commitment, prosocial behavior at work and organizational efficiency and effectiveness [6]. On the one hand, protégés in the mentoring relationship can better understand the organizational goals and have a strong desire to retain membership in the organization [4]. On the other hand, mentoring plays an important part in organizational change and development [19]. For example, the study found that when the organization changes, the mentoring relationship can improve the productivity of employees, adapt to the reorganized working environment, and reduce the stress of job roles [27].

Second, career optimism plays a mediation role in the impact of the mentoring relationship on protégé career commitment. The mentoring relationship helps the protégé’s career development through career function, psychosocial function and role model function. In addition to improving the protégé’s professional knowledge and skills, it is more important to help the protégé integrate into organizational situations and provide promotion opportunities, which improve the protégé’s career optimism. Previous studies have also shown that a successful mentoring relationship can help protégés learn organizational rules, improve their career satisfaction, increase protégés’ salary, establish their positive influence in the organization, and reduce the turnover rate [13]. Employees without the guidance of a mentor are prone to a lack of understanding of the organization and industry and the direction of career planning, and because they are not supported, they lack a positive working mood and their satisfaction with the organization and work is low. As in the previous meta-analysis on career optimism, individuals with high career optimism can experience more job satisfaction and increase organizational commitment [12]. The process of an individual’s career optimism brought about by career success is due to a large amount of energy invested. To obtain more career optimism, it is necessary to maintain or further invest energy. Career-optimistic individuals’ own optimistic charm and their need for social interaction can increase their sense of belonging at work. Thus promoting the improvement of organizational commitment [12].

Third, protean career orientation plays a moderating role in the influence of the mentoring relationship on career optimism. Protean career orientation weakens the positive influence of the mentoring relationship on career optimism, which provides a basis for the unstable positive result of the mentoring relationship. Previous studies have shown that protean career orientation can promote job-seeking behavior [28]. According to the nature of protean career orientation, compared with those with traditional career orientation, individuals with higher protean career orientation have higher career aspirations, tend to actively seek a more suitable working environment, often show a higher level of career exploration behavior, and have higher turnover intention [23]. A higher protean career orientation makes young protégés give a negative evaluation of their career. Most young people are still in the early stage of their careers and are restricted by multiple conditions, such as career experience and economic pressure. Thus, they cannot choose a career that conforms to their own values when they first enter the workplace [21].

The theoretical significance of this study lies in finding the path through which the mentoring relationship can stimulate the protégé’s career optimism and improve organizational commitment, verifying the positive effect of the mentoring relationship, breaking through the scope of previous studies that mostly considered the objective results of the protégé, and discussing the influence on subjective results. Protean career orientation, as the moderating variable of the mentoring relationship, influences career optimism, which provides an explanation for the unsteady results of the positive utility of the mentoring relationship. Although protean career orientation helps to explain why employees tend to plan and manage their careers independently in today’s career environment, at the same time, it is easier for individuals with protean career orientation to choose frequent career mobility instead of competing for increasingly scarce promotion opportunities along the organizational ladder [29]. The dual nature of the career orientation effect provides a new perspective to explain the positive effect of the unsteady mentoring relationship.

### 5.1. Limitations and Future Research

Although this research has a certain degree of theoretical and practical significance, we should also pay attention to some limitations in the research process and strive to improve it in follow-up research. First, the number of samples in this study is limited and cross-sectional, so the causal relationship between variables should be treated with caution. At present, most studies on the mentoring relationship mainly rely on horizontal field research. According to Allen et al., 96% of the studies adopted a field design, and only 5.1% of the studies adopted an experimental design. In addition, in terms of time range, 90.9% of the 176 studies that collected data adopted a cross-sectional design; that is, data were collected at a certain point in time [30]. Therefore, it is urgent to conduct more experiments and longitudinal studies on guidance to determine the causal relationships between guidance and outcome variables. In future research, we should do our best to expand the number of samples and the diversity of respondents’ occupations, pay attention to the time span and use a longitudinal or experimental research design to establish the causal relationship between variables.

Second, other forms of guidance, such as peer guidance and collective guidance, are also worthy of attention. By reducing the hierarchical structure of the organization and adopting a flatter framework, the organization is undergoing increasing reorganization. In peer or group counseling, environmental factors may affect the degree of the protégé’s acceptance of advice, and organizational culture and organizational support for the mentoring relationship may have more influence on the protégé’s acceptance of advice. Therefore, more research should focus on different forms of mentoring relationships, such as peer mentoring, group mentoring and other alternatives to the traditional mentoring relationship. Future research should take into consideration other professions, such as police, teacher and so on. The importance of the mentoring relationship needs to be discussed further.

### 5.2. Implications for Nursing Management

The practical significance of this study lies in the fact that the research results provide a new perspective on the positive effect of the mentoring relationship and are of great significance to new employees in nursing departments and the sustainable development of hospitals. In the construction of mentoring relationships in hospitals, mentors should give high-quality feedback to protégés, improve the feedback environment, reduce the distance of rights, improve the tacit understanding and trust between mentors and protégés, and cultivate the common learning goal orientation, thus bringing constructive and creative results. Prior research shows that psychological empowerment plays an important role in the positive utility and negative utility of the protean career orientation. Therefore, in an uncertain employment environment and career development background, it is particularly necessary to improve psychological empowerment. Hospitals should provide space for nurses to exert their abilities, enhance opportunities to improve their team cooperation ability, clearly define the scope of nurses’ work and rights, and give nurses the right to take a decision. Hospital administrators should clearly convey the vision of the hospital and how the team and individuals can contribute to the vision so that employees can understand that their contributions are working, thus empowering them. The leader should be able to accept the new nurse to complete the task in a way different from that of the leader or colleagues, avoid minor management in the operation process, provide the necessary resources for the nurse and encourage their feedback, suggestions and consultation.

## 6. Conclusions

This study draws the following conclusions: the mentoring relationship can promote the improvement of protégé organizational commitment; protégé career optimism mediates the mentoring relationship and protégé organizational commitment; and the protean career orientation has a moderating effect on the relationship between mentoring relationship and protégé career optimism. When the protégé’s protean career orientation is low, the mentoring relationship has a significant positive impact on protégé career optimism, but when protégé career orientation is high, it has no significant positive impact on protégé career optimism. These conclusions show some implications for nursing management. Hospitals should provide space for nurses to exert their abilities, enhance opportunities to improve their team cooperation ability, clearly define the scope of nurses’ work and rights, and give nurses the right to make decisions.

## Figures and Tables

**Table 1:** Confirmatory factor analysis results o f each test model.

Model	*x* ^2^	*df*	*x^2^/df*	CFI	TFI	RMSEA	SRMR
Four factors (ST, YB, LG, CN)	37.112	21	1.767	0.996	0.993	0.035	0.013
Three factors (ST + YB, LG, CN)	386.313	24	16.096	0.908	0.862	0.155	0.093
Two factors (ST + YB, LG + CN)	586.280	26	22.549	0.857	0.802	0.185	0.130
Single factor (ST + YB + LG + CN)	1058.642	27	39.209	0.737	0.650	0.246	0.117

Note: n = 371; ST = mentoring relationship, YB = protean career orientation, LG = career optimism, CN= organizational commitment.

**Table 2:** Mean, standard deviation and correlation coefficient of variables.

Variable	*M*	*SD*	1	2	3	4	5	6	7
1. Gender	1.75	0.43	-						
2. Age	30.78	9.53	−0.10	-					
3. Education	2.50	0.99	0.11 *	0.57 **	-				
4. Job tenure	2.00	1.40	−0.08	0.75 **	0.38 **	-			
5. Mentoring relationship	3.44	1.01	−0.01	−0.12 *	0.21 **	0.14 **	-		
6. Career optimism	3.64	1.04	0.05	−0.06	0.11 *	−0.09	0.64 **	-	
7. Protean career orientation	3.79	1.01	0.10	0.22 **	0.14 **	0.26 **	0.53 **	0.66 **	-
8. Organizational commitment	3.35	1.19	0.06	−0.01	0.12 *	−0.1	0.56 **	0.49 **	0.42 **

Note: n = 371; * *p* < 0.05, ** *p* < 0.01.

**Table 3:** The results of hierarchical regressions.

Effects Path	*Coefficent*	*SE.*	*t*	*p*
Direct effect of mentoring relationship on career optimism	0.67 **	0.04	15.74	0.00
Direct effect of career optimism on organizational commitment	0.25 **	0.06	4.05	0.00
Direct effect of mentoring relationship on organizational commitment	0.49 **	0.07	7.37	0.00
Total effect of mentoring relationship on organizational commitment	0.66 **	0.05	12.61	0.00
	Effect	BootSE	BootLLCI	BootULCI
Indirect effect of mentoring relationship on organizational commitment	0.17 **	0.05	0.08	0.28

Note: n = 371; ** *p* < 0.01; *coeff* is unstandardized coefficient.

**Table 4:** The results of moderation effect analysis.

Dependent Variable	Conditional Indirect Effect					Moderated Mediation Effect			
	Moderator	Effect	SE	Lower Limit Confidence Interval	Upper Limit Confidence Interval	Index	SE	Lower Limit Confidence Interval	Upper Limit Confidence Interval
Organizational commitment	Low (M − 1SD)	0.49	0.05	0.39	0.59	−0.02	0.01	−0.04	−0.01
	High (M + 1SD)	0.34	0.05	0.23	0.44				

## Data Availability

The data used to support the findings of this study are available from the corresponding author upon request.

## Appendix A

Mentoring relationship questionnaire

1. My mentor takes a personal interest in my career. (Career Support)
2. My mentor helps me coordinate professional goals. (Career Support)
3. My mentor has devoted special time and consideration to my career. (Career Support)
4. I share personal problems with my mentor. (Psychosocial Support)
5. I exchange confidences with my mentor. (Psychosocial Support)
6. I consider my mentor to be a friend. (Psychosocial Support)
7. I try to model my behavior after my mentor. (Role Modeling)
8. I admire my mentor’s ability to motivate others. (Role Modeling)
9. I respect my mentor’s ability to teach others. (Role Modeling)

Protean career orientation questionnaire

1. I am in charge of my own career. (Self-directed)
2. Ultimately, I depend upon myself to move my career forward. (Self-directed)
3. I am responsible for my success or failure in my career. (Self-directed)
4. Where my career is concerned, I am very much “my own person.” (Self-directed)
5. Overall, I have a very independent, self-directed career. (Self-directed)
6. In the past I have relied more upon myself than others to find a new job when necessary. (Self-directed)
7. Freedom to choose my own career path is one of my most important values. (Self-directed)
8. When development opportunities have not been offered by my company, I’ve sought them out on my own. (Self-directed)
9. I’ll follow my own guidance if my company asks me to do something that goes against my values. (Values-driven)
10. In the past I have sided with my own values when the company has asked me to do something I don’t agree with. (Values-driven)
11. What I think about what is right in my career is more important to me than what my company thinks. (Values-driven)
12. It doesn’t matter much to me how other people evaluate the choices I make in my career. (Values-driven)
13. I navigate my own career, based upon my personal priorities, as opposed to my employer’s priorities. (Values-driven)
14. What’s most important to me is how I feel about my career success, not how other people feel. (Values-driven)

Career optimism questionnaire

1. I get excited when I think about my career.
2. Thinking about my career inspires me
3. Thinking about my career frustrates me.
4. It is difficult for me to set career goals.
5. It is difficult to relate my abilities to a specific career plan.
6. I understand my work-related interests.
7. I am eager to pursue my career dreams.
8. I am unsure of my future career success.
9. It is hard to discover the right career.
10. Planning my career is a natural activity.
11. I will definitely make the right decisions in my career.

Organizational commitment questionnaire

1. I am willing to put in a great deal of effort beyond that normally expected in order to help this organization be successful.
2. I talk up this organization to my friends as a great organization to work for.
3. I would accept almost any type of job assignment in order to keep working for this organization.
4. I find that my values and the organizations values are similar.
5. I am proud to tell others that I am part of this organization.
6. This organization really inspires the very best in me in the way of job performance.
7. I am extremely glad that I chose this organization to work for over others I was considering at the time I joined.
8. I really care about the fate of this organization.
9. For me this is the best of all possible organizations for which to work.
